# Challenges of reverse migration in India: a comparative study of internal and international migrant workers in the post-COVID economy

**DOI:** 10.1186/s40878-021-00260-2

**Published:** 2021-11-03

**Authors:** Asma Khan, H. Arokkiaraj

**Affiliations:** 1 Department of Political Science, Jamia Millia Islamia, New Delhi, India; 2 Eastern Europe - Global Area (EEGA) in Leipzig - Halle - Jena / Saxony, Leibniz Science Campus, Leipzig, Germany

**Keywords:** Economic reintegration, Internal migrants, International migrants, Low-skilled, Post-COVID economy, Repatriation, Reverse migration, Migrant mobility, Stigmatization, Wage theft, Gulf countries, India

## Abstract

In India, the major drivers of both internal and international migration are the prevailing unemployment, competitive labour market and enhanced livelihood prospects in the destination state or country. However, the nationwide lockdown and the sealing of inter-state and international borders to control the COVID-19 pandemic triggered the reverse migration of informal migrant workers. This requires the central and state governments to collectively forge strategies to enable their reverse migration and smooth reintegration in the post-COVID economy. In this paper, we have focused on the inter-state migrants in India and returnee migrants only from Gulf countries as they account for two-thirds of Indian migrants living abroad. This study conducted a comparative analysis of the Indian government’s varied approach towards its internal and international migrants during their reverse migration, repatriation and reintegration after the announcement of the lockdown. Firstly, the paper compares the challenges faced by internal and international migrant workers during these stages with the help of in-depth interview data collected from migrants and social workers. Secondly, the varied governmental responses towards their repatriation are discussed. Thirdly, it analyses the obstacles in their economic reintegration to help frame suitable welfare policies for the Indian migrant community.

## Introduction

India has a predominant share of internal migration and is also the top origin country of international migrants (De, 2019; UNDESA, 2020). The Indian Census (2011) data calculated the total number of internal migrants accounting for inter and intra-state movement to be 450 million, an increase of 45% since the Census 2001 (De, 2019). The Economic Survey of India 2017 estimated the inter-state migrant population as 60 million and the average annual flow of migrants between states was calculated at 9 million between 2011 and 2016 (Sharma, 2017). Uttar Pradesh (UP), Bihar, Madhya Pradesh (MP), Rajasthan are among the major origin states, while Delhi, Kerala, Maharashtra, Gujarat, and Tamil Nadu are among the important destination states for these migrant workers (Kamal, 2018).

India has the largest diaspora (18 million) with the number of migrant workers in Gulf countries alone accounting for 8.4 million (Ministry of External Affairs, 2021; UNDESA, 2020). Among the Gulf countries, United Arab Emirates (UAE), Saudi Arabia and Kuwait are the top most favoured destination countries for the Emigration Check Required (ECR) categories from India.^1^ India is also amongst the largest recipient of remittances at USD 78.6 billion (International Organisation for Migration, 2020). In recent years, the low-skilled labour outflows to Gulf countries from relatively poorer states such as UP, Bihar and West Bengal has increased substantially while those from more prosperous states like Kerala, Tamil Nadu, and Karnataka have reduced (Sasikumar & Thimothy, 2015). Poor wage rates in less prosperous states, persistent wage inequalities between regular and casual workers and lack of formal employment opportunities leads to low-skilled and semi-skilled workers migrate overseas to enhance their economic well-being (Karan & Selvaraj, 2008; Sasikumar & Thimothy, 2015). Owing to the infrastructural development and growing demand in important sectors of the Gulf Cooperation Council (GCC) countries, the reliance on low-skilled or semi-skilled workforce (which constitutes around 85% of the total workforce predominantly engaged in construction and service sector) from India and other Asian and African countries will remain high (GIZ and ILO, 2015).

Migration thus gives an optimistic livelihood strategy for migrant workers and their families, contributes to the economic growth of the destination state/country, while the origin state/country benefits from the remittances and the skills acquired during their migration. Migrants from Kerala, a southern state in India, earn high wages as high-skilled workers in the Gulf, allowing them to remit more (Rajan & Zachariah, 2020). Even though labour migration compensates for labour shortages in the destination states/countries, workers employed in the low-skilled, labour-intensive sectors suffer malpractices such as wage-related abuse, working overtime without compensation, lack of social security cover and lack of protection during recruitment and employment (Srivastava, 2013; International Organisation for Migration, 2020). This points to the vulnerable position of these migrant workers which was heightened further during the COVID-19 crisis.

On 24th March 2020, in order to contain the virus, a strict nationwide lockdown was imposed by India with immediate sealing of the inter-state and international borders within four hours of its announcement. This shocked the unprepared migrant workers, both internal and those working abroad. Similarly, with the spread of COVID-19, the Gulf economy was also halted which led to migrant workers being stranded without food, livelihood, safe place to stay and being desperate to return to India. The lack of governmental planning to ensure the well-being of migrant workers within India and abroad led to a “crisis within a crisis”. In this context, this study conducts a comparative analysis of the challenges of reverse migration of India’s internal and international migrant workers. Three major research questions have been covered a) what are the economic and social challenges during their reverse migration, b) what are the varied governmental responses towards the repatriation of both the categories of migrant workers and c) what is the process of economic integration for the reverse migrants. This paper is divided into five parts. First, the introductory part which provides a basic overview of internal migration within India and international migration from India. The second part explains the research methodology and the third consists of the primary findings presented in a comparative manner in accordance with the research questions. The fourth part consists of the analysis which examines the common themes emerging from the experiences of the internal and international migrants from India. The fifth part provides a brief conclusion for this study.

## Research methodology

In order to study the three research questions mentioned above, telephonic interviews with 65 reverse migrants were conducted during May–August 2020 in India. Table 1 presents the profile of the reverse migrants interviewed for this study. Internal migrants belonging to Indian states such as Bihar, UP, MP, Odisha and Chhattisgarh who had returned from the destination states such as Delhi, Gujarat, Karnataka, and Maharashtra were interviewed. Only reverse international migrants from Gulf countries were contacted as a majority of Indian workers are employed in this region. All the workers covered under this study belong to the low-skilled and semi-skilled workers category. A list of contacts of these migrants was prepared with the help of social workers and other researchers working with them. Telephonic interviews, which proved to be the most appropriate method due to COVID-19 lockdown restrictions, with all the migrants have been written as notes and analysed. The snowball sampling method was adopted at a stage where communication was established with some migrants who connected us to other returnees. Further, interviews with labour migration experts and social workers in Delhi and UP, who have worked closely with the migrants, during the lockdown were conducted and guidance from labour migration experts was sought. Interviews were semi-structured and involved key themes covered in the research questions. The interviews were carried out in Tamil and Hindi, which were the preferred languages of the migrants. In order to protect their identities, pseudonyms have been used to refer to the migrants.

**Table 1:** Profile of the interviewees

Reverse Migrants				
Category	Internal Migrants		International Migrants	
**Gender**	Male	33	Male	22
	Female	10	Female	–
**Occupation**	Construction	36	Construction	18
	Hotel	5	Driver	4
	Driver	2	–	–
**Origin State**	Bihar	12	Tamil Nadu	15
	Odisha	5	UP	5
	UP	10	MP	2
	MP	9	–	–
	Chhattisgarh	7	–	–
**Destination State/Country**	Delhi	27	Kuwait	9
	Karnataka	2	Saudi Arabia	4
	Gujarat	6	UAE	6
	Maharashtra	8	Oman	3
**Total**	**43**		**22**	

Further, this research did not attempt to offer representative samples, rather it aimed to provide narratives of the experiences and challenges faced by the migrants during their reverse migration, repatriation and reintegration. The method of narrative analysis was employed to provide a qualitative understanding of the subjective perceptions and experiences of the reverse migrants during the pandemic which may not be adequately captured by statistics. A comparative analysis of the interviews of the internal and international migrants was done to identify the differences and the common challenges faced by the Indian migrant community during a crisis which needs urgent attention during policy making and implementation.

Some of the operational definitions used in this study are as follows. ‘Internal migrants’ refers to inter-state migrant workers who were stranded in different destination states within India. ‘International migrants’ refers to Indians who had migrated to one of the Gulf countries in search of work. The term ‘origin’ and ‘destination’ refers to one’s native place and place of work, respectively. The term ‘reverse migration’ refers to the process of internal and international migrants returning to their place of origin from the destination state/countries. The term ‘economic reintegration’ refers to finding stable employment at one’s origin state. Low-skilled workers includes migrants who are commonly understood as ‘unskilled workers’.

## Findings of the study

This section consists of the primary findings arranged thematically to highlight the various challenges faced during the reverse migration, repatriation and reintegration of both the categories of migrant workers. Under each subheading, first the narratives of the internal migrants are presented followed by those of international migrants. In the next section, a comparative analysis of the findings is done and some common problem areas emerging from the findings have been delineated.

### Economic challenges during reverse migration

Due to the COVID-19-induced lockdown, the working class, especially the low-income migrant workers, have been the worst affected (Pandey, 2020). They were retrenched in large numbers, were rendered unemployed with their wages unpaid in the destination states which forced them to return to their origin states. Lokesh, one of our respondents and a construction worker who returned from Karnataka to Odisha, the lack of employment and wage theft pushed him to return to his native state during the lockdown. Similarly, Mahesh who was working in a hotel when the lockdown was imposed stated:

“I was in Delhi for the past 15 years… During the lockdown I was provided with full salary for March and very less salary for April. The salary for the month of May was unpaid. I cannot survive in Delhi on my savings without any job. So finally, I came back in the month of June to Bihar.”

A few internal migrants reported that they received work under the same employer/contractor after the lockdown but complained of non-payment of wages during the lockdown period. They were forced to return to their villages due to unpaid wages, no place to live with basic facilities such as electricity and water provided by the contractor/employer and no immediate governmental protection. While recalling the plight of these migrant workers, a social worker in Delhi explained how the migrants faced wage theft and retrenchment by their employers when the lockdown commenced, however, when the restrictions eased and industrial work resumed, they were ready to pay the workers. Such instances reveal how the migrant workers were treated as a means to an end and not as citizens whose welfare matters. However, there were a few internal migrants who had stayed back in Delhi, which was their destination state, even during the lockdown period. The reason they reported for not returning was lack of work in their village and that they still hold their jobs in Delhi. They also stated how their employers had arranged for a place to live on the construction premises, took care of the basic facilities like food, electricity and water supply and that they resumed work once the restrictions were lifted.

Similar despicable conditions were experienced by the Indian migrant workers in Gulf countries. There was an urgency to return to India among them caused by large-scale retrenchments due to the unplanned lockdown (Kumar & Akhil, 2021). Hassan, a driver by profession and the only earning member of his household, was one of the many workers retrenched by the private companies in UAE. He was forced to survive on his savings after returning to his native state Tamil Nadu. Wage theft was commonly reported by both internal and international migrants. Bala, a returnee from Oman to Tamil Nadu, reported how his employer did not pay him his due wages, retrenched him and did not even offer to cover the flight expenses to India. Arun, a construction worker, who returned from Kuwait to Tamil Nadu stated his plight:

“Along with me, three were working as helpers in a construction site. Since the lockdown the work was halted, and we were not getting paid. For a few days, the employer gave us food…Then he asked us to return to India as he has no money to take care of us. When we asked for our salary, he threatened us that he would file a false complaint to the police against us if we ask for money…”

However, a few international migrants reported that their employers paid their due wages, arranged for their return, got their Covid tests done and therefore did not face any problem as far as their return to their village was concerned. None of the respondents of this study were a part of trade unions.

### Social challenges during reverse migration

This segment consists of the social challenges faced by migrants before and after the governmental repatriation. The migrants interviewed reported instances of discrimination against them and being viewed as the spreaders of the virus in the destination city/state, during their journey back home, in quarantine facilities and in their villages. Maitheli, who is a wife of a migrant construction worker, experienced stigmatization when returning from Maharashtra to her village in MP a week before the lockdown. She narrated:

“We started our journey before the lockdown in a bus as we had to attend a wedding in March… However due to the news of the spread of Covid, even then people were avoiding interactions with us… some people even placed a cloth to cover their mouth and nose while passing by…”

Rahul returning from Delhi to the state of Bihar reported his experience of caste-based discrimination at the quarantine centre. He explained how people belonging to the higher castes resided on the ground floor with all the facilities while those belonging to the lower castes were kept on the second floor without facilities. Another international migrant reported lack of basic facilities at the quarantine facility in UP and that his family had to provide him with food. This points to the gross neglect of the migrants and puts the entire rationale of quarantine and social distancing into question.

Amanatullah, an international migrant returning from Kuwait to UP reported how even after completing the quarantine period in both the origin and destination states with proper Covid testing done, the villagers, though temporarily, maintained their distance for a few weeks. The interviews revealed that the nature of discrimination in the villages ranged from physical distancing to isolation and hostility which included threats of cutting off supplies of basic necessities to the migrant workers and their families on the basis of mere suspicion of being infected with COVID-19. Dilip, a construction worker returning from UAE to his village in MP, also reported similar discrimination. However, when inquired about his sentiments regarding this, he also blamed the migrant workers for inviting such discrimination:

“Yes some villagers discriminated against us… It felt bad… but even the migrants are at fault as they hide their symptoms, escape the Covid tests and don’t follow the rules so somewhere or the other the villagers rightly set their distance with them since the nature of virus is dangerous...”

At the destination countries, the Indian migrant workers in Kuwait were stripped of their accommodation and were forced to resort to cramped shelters and unhygienic living spaces. Raju, described the despicable situation of Indian workers in Kuwait, where he had been staying under a shed in a nearby car parking space for the 2 weeks along with 150 more workers. Hailing mostly from Indian states like Uttar Pradesh, Odisha and West Bengal, these migrant workers were getting limited support from a voluntary organization of one meal per day.

Since the international migrants had to incur their own travel fare during their repatriation, the interviewed returnees had inculcated a feeling of discrimination when compared to other Indian citizens who could afford the expenses to return to India. Most of them had limited financial resources which were insufficient to bear the cost of accommodation, food and return tickets. This category of migrants have low literacy level and have little to no bargaining power with their employers who retrenched them abruptly and alienated them in a foreign country.

### Migrant workers’ mobility challenges

Initially, the central government was reluctant to rescue the stranded migrants both within and abroad, despite appeals from different stakeholders such as state governments, civil society and trade unions (Desai, 2020; Haider, 2020). Despite the strict mobility restrictions imposed by the government, the distressed internal migrants kept moving on foot or in unsanitary lorries or trucks towards their origin states due to their inability to sustain in the expensive urban areas (Rather & Yousuf, 2020). They faced numerous problems while attempting to cross state borders such as police brutality, grievous injuries with reports of even death due to exhaustion and dehydration (FPJ Bureau, 2020). There was a lack of coordination among the central and state governments resulting in contradictory stances while handling the mass migration (Rather & Yousuf, 2020). At the same time, the employers retrenched the migrant labourers as their businesses were shut due to the lockdown. This resulted in thousands being stranded on various inter-state borders such as Karnataka-Maharashtra and Delhi-Uttar Pradesh (Abi-Habib & Yasir, 2020). Rannvijay**,** a construction worker who returned from Delhi to Bihar, was rendered jobless and due to lack of proper transportation arrangements by the government, he had to travel back independently in a truck to his village. One of the social workers we interviewed described the desperate circumstances that the low-skilled workers had to face in Delhi due to the imposition of the sudden lockdown. He explained:

“Most of the workers stranded on the streets were from low-income groups and belonged to industries such as construction, restaurant, etc. Many workers were stranded on the streets with their families and were rendered jobless. Nobody was there to help them.”

There was an increased pressure from all the stakeholders as several petitions were filed in High Courts and the Supreme Court of India to rescue stranded migrants in various states/countries (Desai, 2020; NH Political Bureau, 2020). After one and a half month of the lockdown, the central government started *Shramik* (workers) special trains and local buses on the request of the state governments. From May 2020 onwards, 4621 Shramik special trains were operated for rescuing both stranded persons and migrants which transported 63.19 lakh (around 6 million) passengers to their origin states (Ministry of Railways, 2020). The Indian Railways allowed only those passengers to travel who were facilitated by the destination state governments. Given the lack of availability of latest data on internal migrants, the Indian government also launched the National Migrant Information System where details of the migrants commuting via the Shramik trains could be maintained for seamless communication between state governments and contact tracing if needed (Karthikeyan, 2020). There was widespread criticism against the central government for making the poor and distressed migrants pay for their ticket despite amassing huge amounts in the PM-CARES Fund established to provide emergency relief during the COVID-19 crisis. Following much confusion and a political tussle between the central and state governments regarding the sharing of travel expenses even when the special trains were running, the state governments later offered to cover their fare (Dhingra, 2020).

Interviews with migrants revealed the difficulties they faced while boarding the special train. Deepak, returning from Delhi to UP, reported that the passengers were not provided with food and water while other respondents reported that they were provided with one meal on a long journey. The train Deepak boarded left him at a place which was 84 kms away from his home. This experience was shared by a few other respondents where they had to cover the remaining distance on their own. Another complexity was related to the online ticket booking process since most of the migrants lacked access to and knowledge about digital technology. The information regarding the Shramik trains was advertised on digital media and the ticket could be booked only in online mode. One of the respondents highlighted how some migrants were unable to return due to their lack of awareness and inability to book the ticket online. Here it is important to emphasise the role played by NGOs and trade unions in the repatriation of migrants either by bus or special train. Yogesh, who returned from Karnataka to Chhattisgarh, described how the migrants who booked the tickets through exploitative agents paid an exorbitant fee:

“Some received help from their family members and friends. But a majority of the workers went back with the help of NGOs, trade unions and their employers. Those who went back with the help of travel agencies and dealers had to pay around 1500-2000 rupees in order to reach home.”

A number of senior academicians and civil society members had pointed towards the ineffective governmental efforts in spreading awareness about the contact details of the designated officials to help with the free online ticket booking and caution them against the exploitative third parties (Counterview, 2020). This resulted in a number of migrants being unable to return on their own or returning late or still walking on foot towards their origin state even when the trains were operating in comparison to those migrants who had the necessary resources and support.

Following appeals from various stakeholders and Indians stuck abroad, especially from the Gulf countries, the central government initiated the Vande Bharat Mission (VBM) on 7th May, 2020. As per the data provided by the MEA, until 11th September, 2020, over 1,385,670 Indian nationals stranded abroad had been repatriated under the VBM (MEA, 2020). MEA provided a list of country-wise and category-wise registration list of stranded Indians in foreign countries (MEA, 2020a). As per this list, Indian workers stranded in the Gulf were the highest amongst other categories requesting for their repatriation*.* As per the latest statistics available on 10th March, 2021, 3.25 million workers had been repatriated from the Gulf (MEA, 2021). The Kuwait government helped in repatriating the undocumented migrants back to India by paying for their amnesty flights and allowing these migrants to re-apply for their visa at a later date (Dutta, 2021).

In order to board a special flight, returnees from Gulf countries under VBM had to afford their own high-cost flight tickets as per the central government guidelines. Kumar, who worked as a driver in Kuwait, discussed his experience of availing the VBM flight during a telephonic conversation with the Indian Embassy in Kuwait as follows:

“First they asked for details like my name, where I worked, whether I am willing to go back home, they asked about the nature of my problem and after gathering the remaining details, they asked me whether I have the money to purchase the flight tickets. If I say ‘yes’ only then they were proceeding the call, if ‘no’ then they (may) disconnect the call. If I say ‘yes’ then they will ask me to undergo a COVID-19 test and fourteen days quarantine. If we agreed, only then they will inform us about the procedure to book the tickets and our name will be noted. Based on this, we can either go home or not.”

The above narration reveals the plight and vulnerability of the stranded Indian workers awaiting repatriation. Further, the guidelines issued by the Ministry of Health & Family Welfare had prescribed 14 days of mandatory quarantine for all international arrivals with the first 7 days to be spent in institutional quarantine (Ministry of Health and Family Welfare, 2020). All the respondents of this study reported undergoing the COVID-19 tests and either institutional or home quarantine. It should be noted that for internal migrants, quarantine facilities and COVID-19 testing were state-sponsored. However, for international migrants, the expenses for institutional quarantine and COVID-19 testing had to be incurred by the passengers themselves (Srivastava, 2020). They could avail exemption from institutional quarantine but only by submitting a negative RT-PCR test result, which was also an expensive test. Thus, the international reverse migrants had to bear a major financial burden during the governmental repatriation and only those who could afford the high travel expenses could easily avail the VBM flights.

### Process of economic reintegration of reverse migrants

Before discussing the experiences of the respondents with regard to their economic reintegration, we will mention the short-term and long-term measures taken by the central government to reintegrate the migrant workers in the post-COVID-19 economy. The central government announced a Rs 1.70 lakh crore (US$ 22.8 billion) relief package for the vulnerable sections which included categories of people who are migrants (Ministry of Finance, 2020). The central government urged the state governments to mobilize the Building and Other Construction Workers (BOCW) Welfare Fund which would benefit around 35 million construction workers registered under the Act (Ministry of Labour and Employment, 2020). However, it should be noted that there are an estimated 56 million workers in the construction sector (Nag and Afonso, 2021)^.^

Additionally, several state governments such as UP, Bihar, Andhra Pradesh and Rajasthan announced one-time immediate cash benefits of Rs 1000 to 5000 (USD 13.59–67.12) and free rations through the Public Distribution System (PDS) (Anand and Thampi, 2020). Subsequently, after immense media attention, another relief package was announced of Rs. 20 lakh crores (USD 270 billion approx.) to benefit the migrant workers, self-employed and small traders. (Ministry of Finance, 2020a). The scheme **‘**One nation one ration card’ was announced to be implemented across India in 2021 to enable migrants to access ration from any fair price shop in India using a digital card. Between April 1 and May 20, 2020, there was a sudden increase in the registrations (around 3.5 million workers) for MGNREGA, a rural employment scheme promising 100 days of work, pointing to increased need for employment (Chauhan, 2020).

It should be noted that such short-term relief packages by the central government were absent in the case of international migrants. Kerala was the only state in India which provided a one-time cash benefit of Rs. 5000 to them (Mathrubhumi, 2020) Also, the Kerala government aimed to help around 5000 Non-Resident Keralites under the Non-Resident Keralites Affairs (NORKA) Department Project for Returned Emigrants (NDPREM) scheme by offering Rs. 50 lakhs (USD 67,123) to each expat to facilitate their own business ventures (ET Bureau, 2020). Acknowledging the huge amount of remittances from the international migrants which benefitted the economy, Kerala also launched an exclusive integration programme called the ‘Dream Kerala Project’. It provides a platform for the business sector to tap the expertise of skilled human resources returning to Kerala after losing jobs abroad (Press Trust of India, 2020). The role of Kerala government in caring for its migrant community from organising community kitchens for stranded migrants to introducing long-term reintegrative measures has been praiseworthy.

As a long-term measure for the labour market integration of both internal and international reverse migrants, the central government announced a Rs 50,000 crore (USD 6.9 billion) ‘Garib Kalyan Rozgar Abhiyan’ which involved skill mapping of migrant workers and connecting women with self-help groups for enhancing employment opportunities. (Ministry of Rural Development, 2020). In view of the lack of data on internal migrants, the government also announced to conduct an All India Survey on Migrant Workers and develop a National Database of Unorganised Workers (NDUW), which would include details of the migrants such as name, occupation, address, educational qualifications and skill type, etc. in order to secure employability and social security benefits for the inter-state migrant workers (Ministry of Labour and Employment, 2021).

SWADES (Skilled Workers Arrival Database for Employment Support), a joint initiative of the Ministry of Skill Development & Entrepreneurship, the Ministry of Civil Aviation and the MEA, aimed to create a database of migrant workers based on their skill set and experience to fulfil the demands of Indian and foreign companies (Ministry of Civil Aviation, 2020). For facilitating employment opportunities, details of SWADES registrations were integrated with Skill India’s ASEEM (Aatmanirbhar Skilled Employee Employer Mapping) portal. As per the latest data, i.e. January 25th, 2021, more than 30,500 workers have registered for the SWADES Skill Card, out of which more than 24,500 are returnees from GCC countries (Ministry of Skill Development and Entrepreneurship, 2021). Further, all data regarding Indians returning under VBM was shared with the state governments.

The internal migrants interviewed reported a sparse coverage of the government relief package as only a few respondents received immediate cash benefits. Only half of the respondents from UP and Bihar received a one-time cash benefit while the remaining did not. Sudesh, a construction worker, reported that he received free ration which would sustain his family only for 15–20 days of a month. A survey of 11,000 migrant workers conducted in April 2020, by SWAN (Stranded Workers Action Network) reported that none of the workers had received ration by the government during the lockdown period (Pandey, 2020). Our study (conducted between May – August, 2020) revealed that half of the respondents were able to avail rations at their native states even though its quantity and duration varied from within and across states included in our study. Those who did not receive free ration reported that they did not have a ration card, or their name was not included in the family’s ration card or were not present to provide a thumb impression to the biometric machine as they migrated to other states hinting at the non-portability of benefits. The data of the Ministry of Consumer Affairs indicated that the free ration scheme had failed because almost 11 states distributed less than 1% of food grains allocated to them (Sharma, 2020). Also, an RTI revealed that barely 10% of the Rs. 20 lakh crore stimulus package was distributed (The Tribune, 2020). Almost all the respondents reported not receiving work under MGNREGA. Ram, a construction worker and a registered MGNREGA worker, who returned to his native state Bihar in June stated that:

“It has been in news that people who have migrated to Bihar shall be provided with employment. But I did not get work under MGNREGA ever since I returned to my village.”

As a result, they were unable to find secure employment in their villages and were willing to remigrate to the urban areas or work under the same contractor/ employer who were unsupportive towards them when the lockdown commenced (Kumar, 2020). Amongst the international migrants interviewed, almost all of them were willing to remigrate abroad once the restrictions eased both in India and at the destination countries. Prem, one of the international migrants from MP, spoke about his desperation to remigrate to cover debts:

“After returning from UAE, so far I did not find any good job opportunities at par with the salary which I was earning abroad. Also our family has some debts which I can only settle if I work abroad for a high salary as the salary is very low here.”

Palani worked as a driver in Saudi Arabia and returned to UP after he was retrenched. However, he was willing to remigrate to any foreign country owing to the low level of wages in India in order to take care of his family. Thus, the data suggests that most of them are eager to remigrate than to stay back in their native states due to low wages, inability to find suitable employment opportunities and governmental support for integration. The remaining migrants, both internal and international, were uncertain about their return due to job losses, closing of the businesses where they used to work at or were willing to begin a new venture in their native states.

## Analysis of the findings

### Forced migrant mobility due to lack of protection by employers and the government

As observed, the worst affected sections of the COVID-19 crisis were the migrant workers, both internal and international, who were pushed to the periphery. Due to the sudden and unplanned lockdown, shutting down of workplaces, hotels, construction work and other sectors which engaged migrant workers, where they earned hand-to-mouth wages, rendered them workless, wageless and homeless. Wage theft is an old phenomena, however the COVID-19-induced migrant crisis magnified this issue (Foley & Piper, 2021). The irresponsibility displayed by the employers, rampant wage thefts and forceful retrenchments in large numbers of both internal and international migrant workers added to their misery (Kuttappan, 2020; Sarkar, 2020). Although the Indian government issued an advisory promising the internal migrants food and shelter, payment of due wages and punitive action against landlords who forcefully evict them but it was rendered ineffective in protecting them.

There are several reasons which add to the precarious circumstances of both the categories of migrant workers. Firstly, they are informal workers with no enforceable contracts between the worker and the contractor. They are not united or backed by trade unions, are less educated, lack knowledge of the job market and good social networks to rely upon in urban areas and foreign countries. This makes them dependent on their contractors and contributes to their lack of bargaining power in case of exploitation (Srivastava, 2013). Describing the work condition of internal migrants*,* the Periodic Labour Force Survey (2017–18) revealed that for more than 70% of the workers in the non-agricultural sector with a regular salary, consisting of mostly migrants, lacked any written job contract and 50% were not enrolled for any social security benefits (Sengupta & Jha, 2020). Also there is unavailability of affordable housing or rental options for the migrants (Iyer, 2020). The exploitative relations between the migrant workers and the employers or sponsors in the kafala system, contract substitution and corruption by middlemen in the Gulf is well-known (Hussain, 2015). In cases of wage theft, most workers are unable to file complaints due to lack of awareness of their rights and costly judicial process. For the international migrants, it is even more complicated to file complaints against their foreign employers after their return to their origin country (Kumar & Akhil, 2021). Therefore, the lack of minimal social and economic protection afforded to migrant workers pushed them back to their village. It can be deduced from our findings that if workers received a safety cover such as a place to live with food, water, free electricity, payment of wages and job security, there is a possibility that they would have stayed back in their work destination. Or the proper arrangement of their return would have contributed to a relatively smoother process of their reverse migration and ensured control over the spread of the virus. However, the rampant unemployment even amongst the general population during the lockdown will further reduce the bargaining power of the migrant workers in the post-COVID economy.

### Migrant experiences of stigmatization, hostility and discrimination

The ill-treatment of the respondents of this study, both internal and international migrants, in terms of being stranded without adequate facilities aggravated their feeling of being discriminated. At the destination state, they were treated as outsiders and a burden when calamity struck with neither the employer nor the government taking their responsibility. The primary findings showed how both the categories of workers were forcefully retrenched and reduced to unsanitary shelters which took away their ability to isolate and put their life at risk. Wage theft and abrupt termination of employment contracts added to the hopelessness of the migrants who incurred huge costs while migrating to Gulf countries in search of a better life (Kumar & Akhil, 2021) Lack of social networks and the hostility and isolation they experienced in the cities, which many migrants referred to as “foreign lands”, forced them to return to their villages (Kumar, 2020a). However, they had to face the social stigma of spreading the virus from the afflicted urban areas by returning to their villages which were relatively protected from the virus at that stage. In the case of internal migrants, the unsanitary conditions under which they returned at a time when travelling was prohibited exacerbated the hostility against them in the form of police brutality and discrimination by villagers. There were instances of social tension among villagers fearing contagion who assaulted the government officials and migrants who were returning (Kumar, 2020; Manoj, 2020). Deplorable condition of health facilities in the rural areas, lack of awareness of the safety measures and proper transportation arrangements by the government also contributed to the fear of the spread of the virus resulting in stigmatisation of the returning migrants. Lack of awareness of government officials in dealing with the returnees was evident when the migrants were sprayed with disinfectants in UP (BBC, 2020). When the government initiated transportation, most of the trains were carrying migrants from COVID-19 hotspots which raised concerns about their isolation (Sheriff et al., 2020). The instances of non-cooperation by migrants needs to be viewed in a larger context of lack of awareness, hostility towards migrant mobility, lack of basic amenities in the destination states and quarantine facilities and a resulting sense of distrust. In order to avoid discrimination and stigma, migrants tend to hide their symptoms, avoid seeking immediate healthcare and observing healthy behaviour (WHO, 2020)*.* The governmental controls over migrant mobility in the name of containing the virus perpetuated discrimination against the migrants who were viewed as vectors of the virus (White, 2020).

### High migration costs for international migrants

Though both the categories of Indian migrant workers faced problems while seeking help from the Indian government, there are some differences which can be pointed out. The international migrants had to incur huge expenses on their ticket, Covid tests and quarantine centres in contrast to the internal migrants whose fare was covered by the respective state governments, even though some internal migrants ended up paying due to exploitative agents, lack of awareness and confusion at the political level. This difference of treatment is due to inadequate media attention and, as a result, lack of governmental attention paid to the concerns of international migrants. It must be highlighted how Indians travelling to foreign countries have to pay an exorbitant recruitment fee especially when travelling through an agent (Migration News, 2008). Such high costs of migration especially amidst a crisis, has the potential to push the blue collar international migrants into major debt and make their reverse migration challenging.

### Inadequate database and lack of inclusive legal and social security for migrants

There is lack of reliable data on internal migrants with the government, unlike international migrants, as the last time any official data was collected was as part of the National Sample Survey 2007–08 and the Census 2011 whose data was partially released in 2020. During the lockdown, the Indian government never collected data on the deaths of internal migrants during their reverse migration and their job losses (Paliath, 2021). The lack of data culminated in the lack of clear direction among the central and state governments on the handling of the migrant mobility and also resulted in their non-coverage of social security measures. The Inter-State Migrant Workmen (Regulation of Employment and Conditions of Service) Act, 1979 aimed to protect migrant workers during their recruitment and transportation against abuse and exploitation by unregistered contractors but it has been poorly implemented (Sen, 2020). Also, in 2020, it has been subsumed under the Occupational Safety, Health and Working Conditions Code, where it is applicable on establishments that employ five or more employees which renders migrants working in micro units outside the ambit of the law. Exclusion, poor implementation, lack of awareness and difficult application process of the governmental schemes providing affordable housing, food and cooking oil subsidies under the public distribution system, affordable public healthcare system renders the low-income migrant communities insecure (International Labour Organisation, 2020).

### Positive role of the civil society

The civil society played a major role in supporting the migrants at all stages of their reverse migration. NGOs in India and in the Gulf countries were at the forefront during the distribution of food and medicines to stranded migrants, directing them to shelter homes, organising bus services, booking tickets of governmental transportation and facilitating undocumented migrants (Business & Human Rights Resource Centre, 2020; Som, 2020). They even conducted migrant surveys and compiled useful reports highlighting the tremendous problems they face generally and during the lockdown. The immense outreach of NGOs with the migrant workers at the grassroot level calls for a concerted effort between the civil society and the government in policy making for migrants.

### Ineffective reintegration measures

Since labour is in the concurrent list, it is important for the central government to set a standard for the other states to follow. In this context, the non-inclusion of international migrants in the relief package was blameworthy. Though international migrants bring in huge remittances benefitting the Indian economy but amidst a crisis, they were left on their own instruments to cover their basic needs, travel fare during repatriation and survival in their origin country. This aspect did not receive enough media attention in comparison to the internal migrants. However, this was not enough to ensure the smooth reintegration of internal migrants as well, as more than 90% of India’s workforce is in the unorganised sector, which includes the low-income migrant workers, who are not registered under welfare schemes, lack wage protection and bank accounts (Express News Service, 2015). This, along with the faulty distribution of the stimulus packages, deprived them of its benefits. Even for those who managed to receive the one-time financial assistance, the amount was extremely small to meet the basic needs for even a month. The continuing pandemic and repeated lockdowns makes the situation for migrants difficult as they are unable to find jobs in their village and face travel restrictions which hinders their job search in urban areas simultaneously. Though skill mapping and maintenance of database are steps in the right direction, it has to be followed up with proper implementation and, most importantly, job creation in the origin states and under rural employment guarantee schemes like MGNREGA. In fact, renowned economist Jean Dreze has touted the idea of a state-sponsored urban employment scheme which will integrate the urban poor (Vij, 2020). A universal social protection cover, not only for the internal migrants but also international migrants upon their return, is important without which any reintegrative measure will remain futile.

This study highlighted the various aspects where the experiences of the internal and international migrants differed as well as converged especially during the pandemic. Some obvious differences were on account of them being separate categories in terms of their work destinations and migration process. However, as far as the differences in terms of the disparate media attention, share in relief package and reintegrative measures, high costs of migration, maintenance of proper database, all these can be commonly attributable to governmental neglect of migrants. The convergences drawn in this study are therefore important to highlight the general vulnerability of the migrants, irrespective of their category, even though both the origin and destination states benefit from migration. Their experiences converged in terms of the lack of planning and protection for the migrant community which led to them being stranded, economic challenges such as wage theft, retrenchments, survival on meagre savings, lack of social security protection, lack of governmental and employer accountability, social discrimination and hostility, mobility issues both before and after repatriation, difficulty in access to justice, ineffective reintegrative measures and vulnerability especially of the low-skilled workers. These commonalities reveal a general precarity of the Indian Migrant community and glaring caveats in migration policy making and implementation. There is a need to work on these aspects to make both internal and international migration a smoother process where all the stakeholders benefit especially in a post-crisis situation.

## Conclusion

This study highlighted the involuntary and forced nature of reverse migration due to the sudden lockdown, lack of preparedness and planning among the government, the irresponsible behaviour of the employers and social hostility against the migrants. Lack of migrant data and registration in welfare schemes excluded most of them from the relief package benefits. The COVID-19 crisis has magnified several pre-existing problems faced by the migrant communities which led them to suffer invariably at different stages of their reverse migration. This crisis, therefore, should be used as an opportunity to bring positive measures and requires strong political will to implement them. The effective reintegration of the internal and international migrants in the post-COVID economy is an important policy issue which would entail collecting latest data, job creation which matches their skill set, inclusion in welfare schemes, portability of social security benefits taking into consideration the mobile nature of migrant communities. The Indian Community Welfare Fund should be mobilized not only in times of crisis but also in reducing the migration costs for international migrants. For better policy making, government must integrate with the civil society which has good outreach with the migrant communities at the grassroot level.

## Data Availability

This research includes primary data collected through interviews of migrant workers and social workers which is available with the authors. This data is not publicly available to protect the privacy of the respondents and only their real occupation, origin and work destination state has been reported in the manuscript. Some of the other data used for supporting key arguments are as follows. These links also feature in the reference list: i. GIZ and ILO., 2015. Labour market trends analysis and labour migration from South Asia to Gulf Cooperation Council countries, India and Malaysia. *Deutsche Gesellschaft für Internationale Zusammenarbeit (GIZ) GmbH and International Labour Organization: Nepal.* https://www.ilo.org/wcmsp5/groups/public/%2D%2D-ed_protect/%2D%2D-protrav/%2D%2D-migrant/documents/publication/wcms_378239.pdf*.* ii. Ministry of External Affairs., 2021. NUMBER OF INDIAN WORKERS IN GULF AND EMIGRATION CHECK REQUIRED (ECR) COUNTRIES*. Government of India.* https://www.mea.gov.in/Images/arebic/ru2653_00.pdf. iii. MEA, 2021. QUESTION NO.2643 INDIANS EVACUATED DURING COVID-19 PANDEMIC. *Government of India*. https://www.mea.gov.in/rajya-sabha.htm?dtl/33666/QUESTION_NO2643_INDIANS_EVACUATED_DURING_COVID19_PANDEMIC. iv. MEA., 2020. QUESTION NO.479 STRANDED INDIANS ABROAD. *Government of India.* https://www.mea.gov.in/lok-sabha.htm?dtl/32978/QUESTION+NO479+STRANDED+INDIANS+ABROAD. v. MEA., 2020a. QUESTION NO.479 STRANDED INDIANS ABROAD. *Government of India.* https://www.mea.gov.in/Images/amb1/lu479_16_09_20.pdf. vi. Ministry of Railways., 2020. Booking in Shramik Special Trains. *Government of India.* http://164.100.24.220/loksabhaquestions/annex/174/AU481.pdf. vii. Sasikumar & Thimothy, 2015. From India to the Gulf region: Exploring links between labour markets, skills and the migration cycle. *Deutsche Gesellschaft für Internationale Zusammenarbeit (GIZ) GmbH & International Labour Organisation (New Delhi, India).* https://www.ilo.org/wcmsp5/groups/public/%2D%2D-asia/%2D%2D-ro-bangkok/%2D%2D-sro-new_delhi/documents/publication/wcms_397363.pdf. viii. UNDESA., 2020. International Migration 2020 Highlights. https://www.un.org/en/desa/international-migration-2020-highlights. ix. International Organisation for Migration. 2020. World Migration Report 2020. *UN Migration*. https://publications.iom.int/system/files/pdf/wmr_2020.pdf.

## Abbreviations

ECR: Emigration Check Required
GCC: Gulf Cooperation Council
MP: Madhya Pradesh
MEA: Ministry of External Affairs
UAE: United Arab Emirates
UNDESA: United Nations Department of Economic and Social Affairs
UP: Uttar Pradesh
VBM: Vande Bharat Mission

## Footnotes

1 According to Emigration Act, 1983 “person whose educational qualification is below matric (10th class) are categorized as ECR passport holders”. They have to obtain an “Emigration clearance” from the concerned office of Protector of Emigrants (POE) before travelling to any of the 18 ECR countries which includes the GCC countries.
